# Knockdown of ADORA2A antisense RNA 1 inhibits cell proliferation and enhances imatinib sensitivity in chronic myeloid leukemia

**DOI:** 10.1080/21655979.2021.2024389

**Published:** 2022-01-16

**Authors:** Yabo Liu, Huibo Li, Yanqiu Zhao, Dandan Li, Qian Zhang, Jinyue Fu, Shengjin Fan

**Affiliations:** Department of Hematology, The First Affiliated Hospital of Harbin Medical University, Harbin, Heilongjiang, China

**Keywords:** Chronic myeloid leukemia, ADORA2A-AS1, miR-665

## Abstract

Long non-coding RNAs (LncRNAs) exert important regulatory roles in chronic myeloid leukemia (CML). In this study, we aimed to investigate the potential role and molecular mechanism of lncRNA ADORA2A antisense RNA 1 (ADORA2A-AS1) in CML. We found that the expression of ADORA2A-AS1 was upregulated in CML. Further, knockdown of ADORA2A-AS1 inhibited the proliferation, induced apoptosis, arrested cell cycle, and enhanced imatinib sensitivity in CML cells. Besides, ADORA2A-AS1 promoted the expression of transforming growth factor-beta receptor 1 (TGFBR1) and ATP binding cassette subfamily C member 2 (ABCC2) via sponging miR-665, thereby exerting a tumor-promoting activity. Collectively, our results confirmed the oncogenic effect of ADORA2A-AS1 in CML, indicating that ADORA2A-AS1 is a promosing therapeutic target for CML.

## GRAPHICAL ABSTRACT

**Figure:**
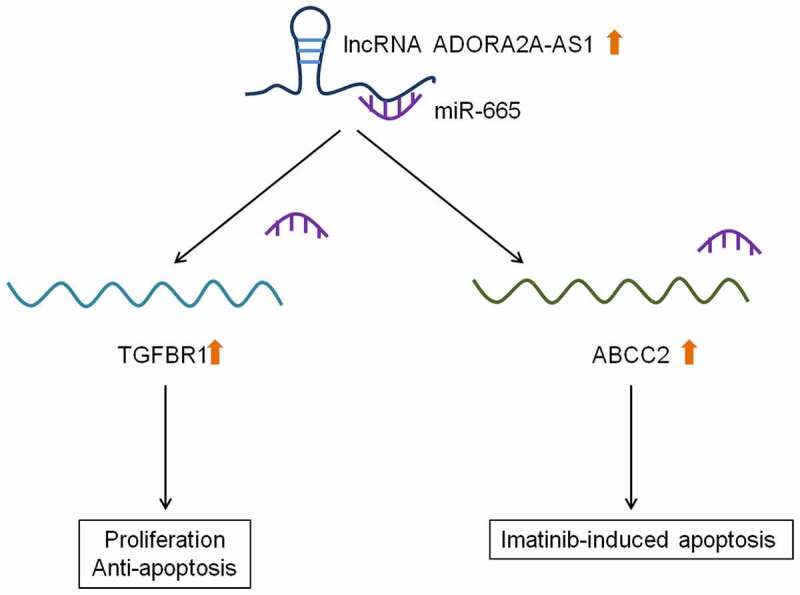

## Background

Chronic myeloid leukemia is a malignant clonal hematopoietic stem cell disease that accounts for about 20% of adult leukemia [1]. CML is characterized by the BCR-ABL fusion gene formed by the translocation of the ABL gene on chromosome 9 to the BCR gene on chromosome 22. The protein encoded by the BCR-ABL gene has strong tyrosine kinase activity, which disrupts the normal function of the hematopoietic system, inhibits normal cell apoptosis, causes the disaffected cells to gather in the hematopoietic tissue such as bone marrow, and inhibits the normal hematopoietic function [2]. The application of BCR-ABL tyrosine kinase inhibitor imatinib improves the prognosis of patients with CML [3]. However, the resistance of tumor cells to imatinib severely limits its clinical application [4]. Current studies on the occurrence of secondary drug resistance include amplification and increased expression of BCR-ABL fusion genes, multidrug resistance gene overexpression, and drug resistance of CML stem cells. Therefore, it is of great value to identify the key factors influencing the mechanism of drug resistance and the development of combination drugs.

LncRNAs are a class of non-coding transcripts with a length of more than 200 nucleotides, which constitute a large part of the human gene transcriptome. Studies have reported that lncRNAs regulate neighboring or distant genes by serving as signaling, guiding, isolating, or scaffolding molecules [5]. LncRNAs act as oncogenes or tumor suppressor in cancers [6,7]. LncRNAs are frequently dysregulated in BCR-ABL-mediated CML [8]. G Guo et al. [9] identified that lncRNA BGL3 regulated BCR-ABL-mediated malignant transformation. Juhua Yang et al. [10] showed that H19 was underexpressed in CML, while overexpression of H19 reduced cell proliferation in CML cell lines and prolonged survival in the xenografted mouse. Zhang F et al. [11] revealed that lncRNA FENDRR restricted the expression of MDR1 via interacting HuR and miR-184 in CML cells, thereby reducing adriamycin resistance. ADORA2A-AS1 is a lncRNA located on human chromosome 22p15.5. Previous studies have reported that ADORA2A-AS1 was associated with dietary habits [12]. However, the role of ADORA2A-AS1 in CML progression remains unclear.

In this study, we first analyzed the expression trend of ADORA2A-AS1 in CML patient samples. Then, RNA interference technique was used to knock down ADORA2A-AS1 in CML cells to study the changes of CML cell viability, apoptosis, cell cycle and sensitivity to treatment. In addition, bioinformatics prediction and experimental techniques were used to explore the mechanism of ADORA2A-AS1 in CML cells. This experiment will elucidate the occurrence and development of CML at the epigenetic level, thus providing potential intervention targets for its treatment.

## Materials and methods

### Human samples

Bone marrow samples were collected from 13 patients with CML and 8 healthy controls at Department of Hematology of the First Affiliated Hospital of Harbin Medical University from June 2021 to December 2021. CML was confirmed by clinical examination, cell morphology analysis, immunology, and histochemical staining. Samples were collected from all patients prior to any therapeutic intervention. Patients were divided into the CP phase (8 cases) and AP phase (5 cases), and the characteristics of the patients were listed in (Table S1). Ten healthy volunteers included 6 males and 4 females, with an average age of 36.4 (35.6–56.8). This study was approved by the Ethics Committee of the First Affiliated Hospital of Harbin Medical University. All participants signed written informed consent before the study.

## Cell lines and culture conditions

The K562, KCL22 and HEK-293 T cells were obtained from the Cell Bank of the Chinese Academy of Sciences (Shanghai, China). The KCL22 cells were maintained in Iscove’s modified Dulbecco’s medium (IMDM; Thermo Fisher Scientific, Shanghai, China) supplemented with 10% fetal bovine serum (FBS). The K562 and HEK-293 T cells were cultured in RPMI 1640 medium (Gibco, USA) containing 10% FBS. All cells were incubated in a humidified incubator with 5% CO_2_ at 37°C.

## Western blot

The CML cells were lysed with RIPA Lysis Buffer (Beyotime, Shanghai, China) and then centrifuged. The supernatant was collected and stored at −20°C. Protein concentration was measured with a Bicinchoninic Acid Protein Assay Kit (Beyotime, Shanghai, China). After the mixture with loading buffer, 50ng protein was separated in 10% sodium dodecyl sulfate-polyacrylamide gel electrophoresis gel and then transferred to an activated polyvinylidene fluoride membrane (Millipore, Billerica, MA, USA). The membrane was sealed in the blocking solution at room temperature for 10 min and incubated with the diluted primary antibody overnight at 4°C. After washing 3 times, the membrane was soaked in secondary antibody solution for 1 h. Finally, enhanced chemiluminescence (ECL, Thermo Fisher, Waltham, MA, USA) was performed to visualize protein bands, and Image J software (NIH, Bethesda, MD, USA) was used for density analysis, and glyceraldehyde-3-phosphate dehydrogenase (GAPDH) was used as an internal reference to quantify protein expression levels. Antibodies against TGFBR1 and ABCC2 were purchased from Abcam (Shanghai, China). GAPDH monoclonal antibody was obtained from Proteintech (Wuhan, China)

## Quantitative real-time polymerase chain reaction (qRT-PCR)

For CML cells or samples, total RNAs were extracted with TRIzol Reagent (Invitrogen, Carlsbad, CA, USA). The extracted RNAs were then reversed to cDNA using the EasyScript One-step gDNA Removal and cDNA Synthesis Supermix kit (TransGen Biotech, Beijing, China). Quantitative real-time PCR was performed with an SYBR green PCR kit (Vazyme, Nanjing, China). Primer sequences are provided in (Table S2). GAPDH was used as an internal control for ADORA2A-AS1. U6 was used as an internal control for miR-665. The relative expression levels of the transcripts were calculated using 2^–ΔΔCt^.

## Cell proliferation assay

Cell proliferation was measured by CCK-8 Cell Proliferation and Cytotoxicity Assay Kit (Solarbio, Beijing, Jiangsu, China) and Cell-Light EdU DNA Cell Proliferation Kit (RiboBio, Shanghai, China). For CCK8 assay, the transfected CML cells (5 × 10^3^ cells/well) were seeded into 96-well plates and then cultured for 0, 24 h, 48 h and 72 h 10 μL of CCK-8 reagent was added and incubated for 3 h. Lasty, the absorbance was measured at 450 nm. For EdU assay, 5 × 10^3^ cells were seeded into 96-well plate and cultured for 48 h. Then, cells were dyed with EdU reagent for 2 h and stained with DAPI solution. The images was collected with a fluorescence microscope (Olympus, Japan)

## Flow cytometry

Cell cycle and cell apoptosis were evaluated by flow cytometry following previous studies [13]. The CML cells were resuspended in 500 μL of binding buffer, incubated with 5 μL of Annexin V-FITC and 5 μL of PI for 10 min, and analyzed using flow cytometer. The Annexin V-positive cells were identified as apoptotic cells.

## Luciferase reporter assay

Luciferase reporter assay was carried out according to previous reports [14]. The luciferase reporter constructs containing the wild-type and mutated 3`-UTR fragment of ADORA2A-AS1 (ADORA2A-AS1-WT and ADORA2A-AS1-MUT) were obtained from GeneChem (Shanghai, China). HEK-293 T cells were co-transfected with ADORA2A-AS1-WT or ADORA2A-AS1-MUT luciferase reporter, and miR-665 mimics or mimics control, using Lipofectamine 2000 reagent (Invitrogen). After 48 h of transfection, cells were measured with the Luciferase Reporter Assay System (Promega).

## RNA immunoprecipitation (RIP) assay

RIP experiment was performed according to previous studies [15]. Magna RNA-Binding Protein Immunoprecipitation Kit (Millipore, Bedford, MA, USA) was used for RIP assay. Briefly, after reaching 80%-90% confluency, cells were collected and lysed in RIP lysis buffer. Afterward, the whole-cell extract (100 μL) was coimmunoprecipitated with prepared RIP buffer. Then, the mixture was digested with proteinase K, and then immunoprecipitated RNAs were isolated, purified, and subjected to qPCR analysis of ADORA2A-AS1 and miR-665. The RNA levels were normalized to the input RNA levels.

## Statistical analyses

All statistical analyses were conducted using GraphPad Prism Software, version 8.0 (San Diego, USA). The results are expressed as mean ± standard error of the mean (SEM) based on at least three independent replicates. Student’s t-test or one-way analysis of variance was used for two or multiple group comparisons. *P* < 0.05 was considered statically significant.

## Results

In this study, we aimed to investigate the role and mechanism of lncRNA ADORA2A-AS1 in CML. We first used qRT-PCR to examine the expression of ADORA2A-AS1 in CML and healthy control. Then, the effects of silencing ADORA2A-AS1 on cell proliferation, apoptosis and cell cycle were evaluated in a series of cell experiments. Furthermore, the potential miRNAs regulated by ADORA2A-AS1 were predicted and verified by RIP and luciferase assay. Rescue experiments confirmed that miR-665 could reverse the carcinogenic effects of ADORA2A-AS1. Besides, TGFBR1 and ABCC2 were identified as the targets of ADORA2A-AS1/miR-665 and meadiated their role in CML.

## LncRNA ADORA2A-AS1 is overexpressed in CML

**Figure 1.**
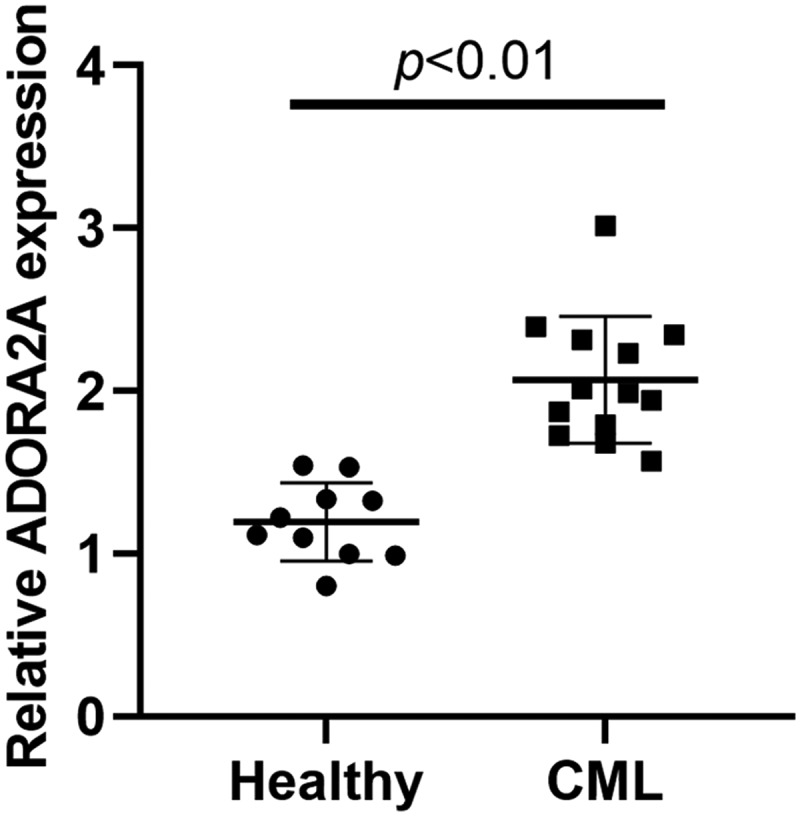
The expression of ADORA2A-AS1 is upregulated in CML. The expression of ADORA2A-AS1 in CML patients (n = 13) and healthy individuals (n = 10) by qRT-PCR.

The expression of ADORA2A-AS1 was examined in bone marrow samples from CML patients (n = 13) and healthy individuals (n = 10) by qRT-PCR. The results revealed that the levels of ADORA2A-AS1 were dramatically increased in CML patient samples compared with those in healthy individuals (Figure 1).

## Knockdown of ADORA2A-AS1 suppresses CML cell proliferation

**Figure 2.**
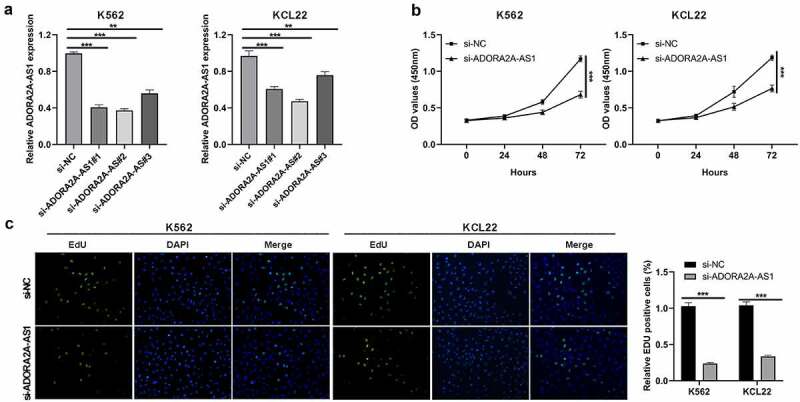
Knockdown of ADORA2A-AS1 inhibits cell proliferation. (a) Transcript levels of ADORA2A-AS1 in K562 and KCL22 cells transfected with control siRNA or ADORA2A-AS1 siRNA. (b) Representative CCK8 proliferation assays in K562 and KCL22 cells. (c) Representative EDU staining (left) and quantitation (right) in K562 and KCL22 cells. **P < 0.01, ***P < 0.001.

Given that ADORA2A-AS1 expression was elevated in CML, we wondered whether ADORA2A-AS1 affects cellular biology. Three siRNAs targeting ADORA2A-AS1 were designed to silence its expression, and si-ADORA2A-AS1#2 showed a better silencing efficiency in both K562 and KCL22 (Figure 2(a)) and was chosen for the subsequent experiments. CCK8 assay demonstrated that OD values of ADORA2A-AS1-silenced cells via si-ADORA2A-AS1 transfection were lower than negative control cells (Figure 2(b)). EdU staining assay showed that EdU incorporated cell proportion of si-ADORA2A-AS1-transfected cells via si-ADORA2A-AS1 transfection were lower than si-NC-transfected K562 and KCL22 cells (Figure 2(c)). The two assays revealed that silencing ADORA2A-AS1 inhibted cell proliferation of CML cells.

## Knockdown of ADORA2A-AS1 induces cell apoptosis and cell cycle arrest

**Figure 3.**
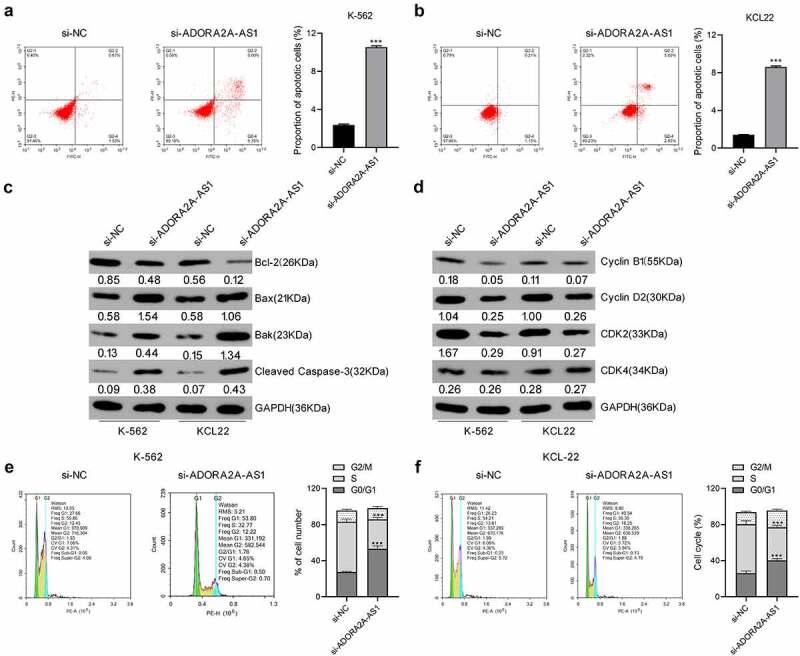
Knockdown of ADORA2A-AS1 induces CML cell apoptosis and cell cycle arrest. Cell apoptosis assay in K562 (a) and KCL22 (b) cells, analyzed by flow cytometry. (c) Cell apoptosis-related proteins, measured by Western blot. (d) Cell cycle-related proteins, measured by Western blot. Cell cycle analysis in K562 (e) and KCL22 (f) cells, analyzed by flow cytometry. ***P < 0.001.

To further explore whether exhaustion of ADORA2A-AS1 influences cell apoptosis and cell cycle in CML, flow cytometry analysis was performed. As described in (Figure 3(a,b)), compared to control cells, the proportion of apoptotic cells was increased in si-ADORA2A-AS1-transfected K562 and KCL22 cells. Meanwhile, inhibition of ADORA2A-AS1 increased the levels of pro-apoptotic proteins Bax, bak, and cleaved caspase-3 and inhibited the levels of anti-apoptotic protein Bcl-2 in K562 and KCL22 cells (Figure 3(c)). These data indicated that knockdown of ADORA2A-AS1 promotes CML cell apoptosis. Also, knockdown of ADORA2A-AS1 caused cell arrest in G0/G1 phase (Figure 3(e,f)). Consistently, the protein levels of cyclin B1, cyclin D2, and CDK2 were decreased after ADORA2A-AS1 knockdown in CML cells (Figure 3(d)).

## ADORA2A-AS1 acts as an endogenous sponge for miR-665

**Figure 4.**
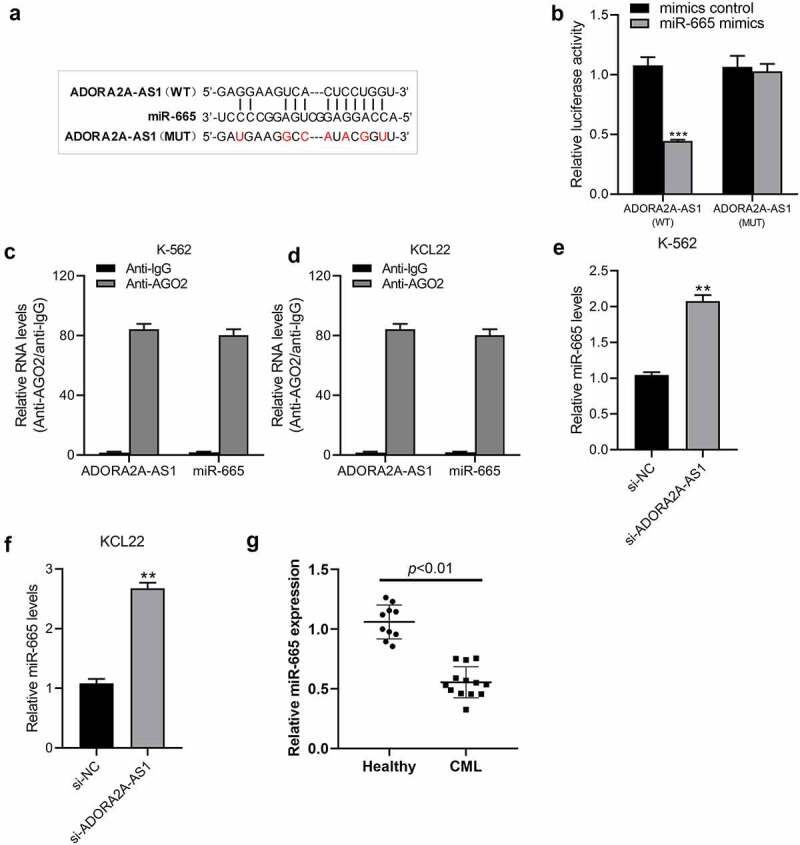
ADORA2A-AS1 acts as an endogenous sponge for miR-665. (a) The predicted binding sequence of miR-665 on ADORA2A-AS1, and the mutants of ADORA2A-AS1 and miR-665. (b) The relative luciferase activity in HEK 293 T cells cotransfected with ADORA2A-AS1-WT or ADORA2A-AS1-MUT reporter and miR-665 mimics or mimics control. RIP assay was performed in K562 (c), and KCL22 (d) cell extracts to examine miR-665 endogenously associated with ADORA2A-AS1. qRT-PCR was used to examine the expression of miR-665 in K562 (e) and KCL22 (f) cells transfected with si-ADORA2A-AS1 or si-control. (g) The expression of miR-665 in healthy donors and CML patients. **P < 0.01, ***P < 0.001.

The potential miRNAs interacting with ADORA2A-AS1 were analyzed using the online database starBase 3.0 (http://starbase.sysu.edu.cn/) [16], and miR-665 was predicted to be the potential target of ADORA2A-AS1 (Figure 4(a)). To further verify the interaction between ADORA2A-AS1 and miR-665, we constructed luciferase reporters containing wild-type or mutated ADORA2A-AS1 sequence that harbored potential binding sequence for miR-665, and a dual-luciferase reporter assay was then carried out. As illustrated in (Figure 4(b)), transfection of HEK-293 T cells with miR-665 mimics failed to reduce the luciferase activity of the ADORA2A-AS1-MUT, but significantly reduced luciferase reporter activity of ADORA2A-AS1-WT. LncRNA interacts with miRNAs through the Ago2-containing RNA-induced silencing complex (RISC). To confirm the interaction of ADORA2A-AS1 and miR-665 at endogenous levels, a RIP assay was conducted to pull down endogenous lncRNAs and miRNAs in CML cells using the antibody against Ago2. The results showed that ADORA2A-AS1 and miR-665 were both concurrently enriched in Ago2 pellets of K562 and KCL22 cells extracts relative to the IgG control group(Figure 4(c,d)), indicating the mutual interaction between ADORA2A-AS1 and miR-665. Furthermore, knockdown of ADORA2A-AS1 increased the expression of miR-665 in K562 (Figure 4(e)) and KCL22 (Figure 4(f)) cells. In addition, we detected the expression of miR-665 in healthy donors and CML patients and found that the levels of miR-665 were significantly lower in CML than those in healthy donors (P < 0.001) (Figure 4(g)). Together, these results implied that ADORA2A-AS1 acts as an endogenous sponge to suppress miR-665 levels.

## miR-665 counteracts the oncogenic activity of ADORA2A-AS1 in CML cells

**Figure 5.**
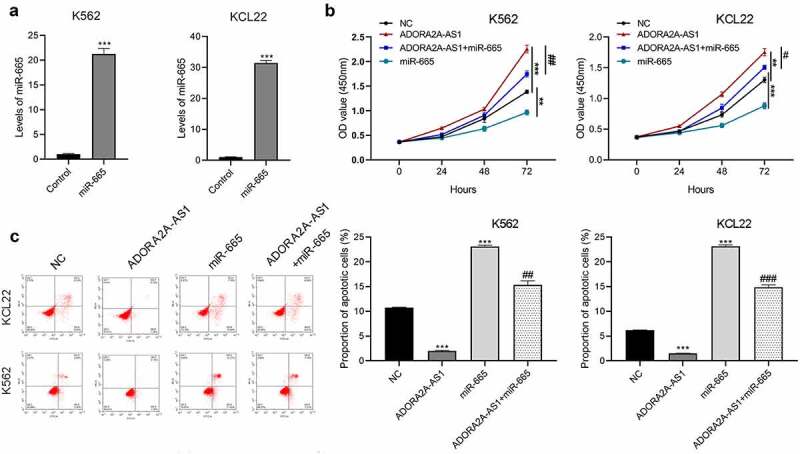
miR-665 counteracts the malignant properties of ADORA2A-AS in CML cells. (a, b) The expression of miR-665 in K562 and KCL22 cells transfected with miR-665 mimics or mimic control. K562 and KCL22 cells were co-transfected with miR-665 mimic and pcDNA3.1-ADORA2A-AS or alone, and subjected to (c, d) CCK8 assay for cell proliferation, (e) flow cytometry for cell apoptosis analysis, and (f) flow cytometry for cell cycle analysis. *P < 0.05, **P < 0.01, ***P < 0.001;. #*P* < 0.05, ##*P* < 0.01.

Commercial miR-665 mimic were transfected into K562 and KCL22 cells to increase miR-665 expression. As shown in (Figure 5(a)), miR-665 mimic efficiently increased the expression of miR-665 in K562 and KCL22 cells compared to the mimic control groups. CCK8 assays showed that overexpression of ADORA2A-AS1 significantly promoted cell proliferation, while miR-665 mimic inhibited cell viability and partially attenuated the pro-proliferative effect of ADORA2A-AS1 in CML cells (Figure 5(b)). Moreover, flow cytometry analysis revealed that overexpression of ADORA2A-AS1 inhibited cell apoptosis, while miR-665 mimic promoted cell apoptosis and also reversed anti-apoptotic effect of ADORA2A-AS1 overexpression in CML cells (Figure 5(c)). Together, these results indicate that miR-665 counteracts the oncogenic activity of ADORA2A-AS in CML cells.

## ADORA2A-AS1 promotes TGFBR1 and ABCC2 expression by adsorbing miR-665

**Figure 6.**
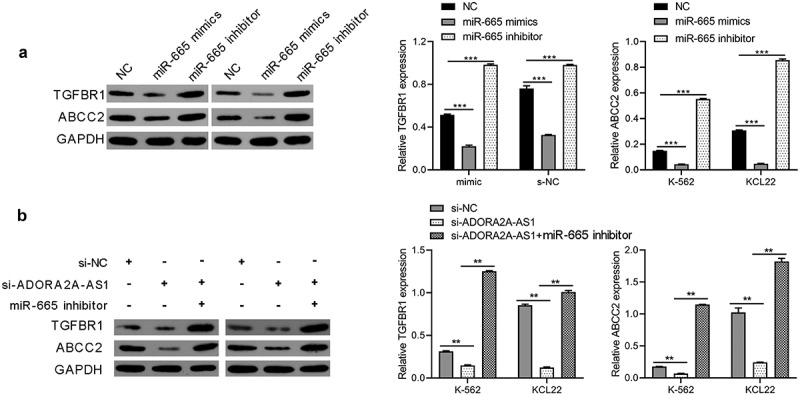
ADORA2A-AS1 promotes TGFBR1 and ABCC2 expression by adsorbing miR-665. (a) Western blot was used to determine TGFBR1 and ABCC2 protein levels in K562 and KCL22 cells transfected with miR-665 mimics or inhibitors. (b) Western blot was performed to determine TGFBR1, and ABCG1 protein levels in K562 and KCL22 cells transfected with miR-665 mimics si-ADORA2A-AS1 or combination. ***P < 0.001.

Previous studies have reported that TGFBR1 and ABCC2 were the direct targets of miR-665 in other cancers [17–19]. Therefore, we investigated whether miR-665 also regulated TGFBR1 and ABCC2 in CML. After transfection with miR-665 mimics or inhibitors, TGFBR1 and ABCC2 proteins in K562 and KCL22 cells were detected by Western blot. The results showed that overexpression of miR-665 inhibited the expression of TGFBR1 and ABCC2, while inhibition of miR-665 promoted the expression of TGFBR1 and ABCC2 (Figure 6(a)). Further, we found that knockdown of ADORA2A-AS1 inhibited the expression of TGFBR1 and ABCC2, which could be reversed by miR-665 inhibitor (Figure 6(b)). These data indicate that ADORA2A-AS1 upregualtes the expression of TGFBR1 and ABCC2 by competitively absorbing miR-665.

## ADORA2A-AS1 affectes imatinib sensitivity

**Figure 7.**
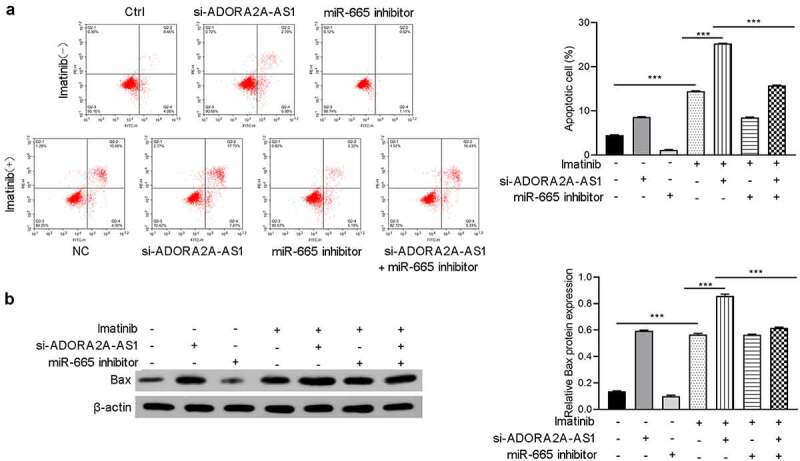
ADORA2A-AS1 affected imatinib sensitivity. After transfection with si-ADORA2A-AS1, miR-665 mimics or combination, K562 cells were treated with imatinib. (a) Cell apoptosis analyses were performed in each group. (b) Western blot was performed to detect Bax protein expression. ***P < 0.001.

Previous studies reported that ABCC2 was involved in the imatinib sensitivity of CML And the above experiments indicate that ADORA2A-AS1 and miR-665 can regulate the expression of ABCC2. Therefore, we next explored whether ADORA2A-AS1 and miR-665 affects the imatinib sensitivity in CML cells. The results demonstrated that knockdown of ADORA2A-AS1 enhanced imatinib-induced apoptosis of K562 cells, while miR-665 inhibitor suppressed imatinib-induced apoptosis and attenuated the pro-apoptotic effect of ADORA2A-AS1 silencing (Figure 7(a)). Moreover, Western blot showed that the expression of Bax was increased after ADORA2A-AS1 knockdown in K562 cells, whereas downregulation of miR-665 exerted the opposite effect in K562 cells. Rescue experiments were performed to confirm that miR-665 inhibitor reversed the effect of ADORA2A-AS1 knockdown on Bax protein (Figure 7(b)).

## Discussion

At present, tyrosine kinase inhibitors (TKIs) are still one of the main therapies for the treatment of CML, which greatly improves the survival rate of patients with CML by targeting the abnormal BCR-ABL [20]. However, approximately 40% of patients with CML require alternative therapy due to BCR/ABL gene mutations causing resistance to TKIs or drug-related toxicity [21]. In this case, the development of new targets for the treatment of CML may be a good alternative or complement to the traditional approach.

Emerging evidence has shown that abnormally expressed lncRNAs exert key regulatory roles in the occurrence, development, and drug resistance of CML [22–24]. For example, lncRNA H19 was repressed in CML, and overexpression of H19 suppressed cell viability and colony formation in CML cell lines and prolonged survival in xenografted mouse models [10]. LncRNA nuclear-enriched abundant transcript 1 (NEAT1) was downregulated in CML patient samples, and inhibition of NEAT1 promoted imatinib-induced apoptosis [24]. LncRNA FENDRR inhibited MDR1 expression via interacting with HuR and miR-184 and thereby attenuates adriamycin resistance in CML cells [11]. Additionally, MALAT1, MEG3, SNHG5, and HOTAIR have also been reported to modulate imatinib-induced apoptosis in CML cells [5,9–11]. In the present study, we found that ADORA2A-AS1 expression was upregulated in CML samples and cells, and knockdown of ADORA2A-AS1 could inhibit cell proliferation and induce cell apoptosis and cell cycle arrest in CML cells. Moreover, knockdown of ADORA2A-AS1 also enhanced imatinib-induced apoptosis. To the best of our knowledge, this is the first study indicating the oncogenic properties of ADORA2A-AS1 in CML.

Now that we have identified the role of ADORA2A-AS1 in CML, it is of great significance to understand its potential mechanism for further discovering new CML targets and improving the treatment of CML. Previous studies have confirmed that lncRNAs sponge miRNAs to regulate the expression of miRNA target genes [25]. In present study. miR-665 was identified as a direct target of ADORA2A-AS1. As a key regulator, miR-665 has been reported to act tumor-suppressing or oncogenic activities in many kinds of cancers, including gastric cancer, ovarian cancer, breast cancer, and glioma [26–29]. Many important functional genes, including Akt3, STAT3, YAP1, VEGFA, CRIM1, have been identified to bind to and be regulated by miR-665. Interestingly, TGFBR1 and ABCC2 were both identified as target genes of miR-665 in other tumors [30], have also been confirmed to be regulated by miR-665 in CML cells. TGFBR1, as a receptor for TGF-β ligands, plays an important signaling function in the TGF-β signaling cascade. TGF-β pathway has been reported to maintain the transformed progenitor cell population and cell proliferation in CML [31]. TGFBR1 is often overexpressed in multiple malignancies, and downregulation of TGFBR1 inhibited cell growth and metastasis [32,33]. Our study found that knockdown of ADORA2A-AS1 decreased the expression of TGFBR1 by releasing miR-665, which explains reduced proliferation in CML cells after ADORA2A-AS1 knockdown. Another target of miR-665, ABCC2, is a member of the ATP-binding cassette transporter superfamily and is often involved in multidrug resistance in various kinds of cancers. Previous studies have shown that ABCC2 expression was up-regulated in the isolated peripheral blood cells of the CML patients and is associated with imatinib resistance in CML [34,35]. In this study, we found that the expression of ABCC2 was positively regulated by ADORA2A-AS1 and negatively regulated by miR-665. Further, our results confirmed that knockdown of ADORA2A-AS1 promoted imatinib-induced apoptosis, and miR-665 inhibitor could reverse this effect.

The specific expression of lncRNA in CML provides the possibility for its clinical application. Several lncRNAs have been defined as biomarkers, and some of them are undergoing clinical trials [36,37]. In our study, overexpression of ADORA2A-AS1 in CML suggests that it may be used as a marker of diagnosis and prognosis, but its clinical application needs to be verified in more clinical samples. On the other hand, lncRNAs are also potential targets for cancer treatment. Currently, there are several methods, including specific siRNA, antisense oligonucleotides and Gapmers, which can target lncRNAs to regulate their expression, or use aptamers to disrupt the function of lncRNAs. In this study, specific targeting ADORA2A-AS1 significantly restricted the malignant proliferation of CML cells. This suggests that targeted CML therapy based on ADORA2A-AS1 is promising. However, these technologies are still in their infancy, requiring in vivo validation, further development of experimental strategies, and many clinical trials before success.

## Conclusion

Our study demonstrated that ADORA2A-AS1 is upregulated in CML, and knockdown of ADORA2A-AS1 inhibits cell proliferation, induces cell apoptosis and cell cycle arrest, and enhances imatinib-induce apoptosis through regulating miR-665, with TGFBR1 and ABCC2 as its downstream effectors. Together, these findings suggest that targeting ADORA2A-AS1 may represent a novel and important therapeutic strategy for CML.

## Supplementary Material

Supplemental Material

## Data Availability

The datasets used and/or analyzed during the current study are available from the corresponding author on reasonable request.

## Highlights

- High levels and of ADORA2A-AS1 was found in CML.
- Silenced ADORA2A-AS1 inhibits CML progression and enhanced imatinib sensitivity.
- ADORA2A-AS1 upregulates TGFBR1 and ABCC2 expression by adsorbing miR-665.
