# Supplementary material for: Knockdown of ADORA2A antisense RNA 1 inhibits cell proliferation and enhances imatinib sensitivity in chronic myeloid leukemia
Source: Bioengineered. 2022 Jan 16;13(2):2296–307. doi: 10.1080/21655979.2021.2024389 (PMC8973732; doi:10.1080/21655979.2021.2024389)
Supplement: Supplemental Material [file KBIE_A_2024389_SM8348.zip › supplementary/Table_S1.docx]

**Table S1 Characteristics of the patients included in the study.**

| Item | CML-CP （n=8) | CML-AP （n=5) |
| --- | --- | --- |
| Age (years), median（range) | 42 (17-67) | 48 (34-64) |
| Male/female, (n/n) | 4/4 | 3/2 |
| WBCs × 10^9^/median (range) | 76.4 (26.2–196.3) | 34.6 (8.7-193.2) |
| Hemoglobin level (g/l) | 94 (68–126) | 86 (62-126) |
| Platelet count,10^9^/median (range) | 359 (106–597) | 654 (156-864) |
| Bcr/abl^p210^, +/- | 8/0 | 5/0 |
| Bcr/abl^p190^, +/- | 2/6 | 3/2 |

CML, chronic myeloid leukemia; CP, chronic phase; BP, blast phase; WBCs, white blood cells.
