# Supplementary material for: Knockdown of ADORA2A antisense RNA 1 inhibits cell proliferation and enhances imatinib sensitivity in chronic myeloid leukemia
Source: Bioengineered. 2022 Jan 16;13(2):2296–307. doi: 10.1080/21655979.2021.2024389 (PMC8973732; doi:10.1080/21655979.2021.2024389)
Supplement: Supplemental Material [file KBIE_A_2024389_SM8348.zip › supplementary/Table_S2.docx]

**Table S2 The sequences of primers used in this study.**

| **Gene** | **The sequences of primers** |
| --- | --- |
| LncRNA ADORA2A-AS1 | Forward: 5′-CCTGGAGCTCGGATGTGGAA-3′  Reverse: 5′-CCTTTGAACAGGGCTCAGGAC-3′ |
| GAPDH | Forward: 5′-GTCGGAGTCAACGGATTTG-3′  Reverse: 5′-TGGGTGGAATCATATTGGAA-3′ |
| Hsa-miR-665 | Forward: 5'-GGTGAACCAGGAGGCTGAGG-3'  Reverse: 5'-CAGTGCAGGGTCCGAGGTAT-3' |
| U6 | Forward: 5′-CGCTTCGGCAGCACATATAC-3′  Reverse: 5′-TTCACGAATTTGCGTGTCATC-3′ |
